# Testing the Low-density Hypothesis for Reversed Sex Change in Polygynous Fish: Experiments in *Labroides dimidiatus*

**DOI:** 10.1038/srep04369

**Published:** 2014-03-13

**Authors:** Tetsuo Kuwamura, Tatsuru Kadota, Shohei Suzuki

**Affiliations:** 1 School of International Liberal Studies, Chukyo University, Nagoya 466-8666, Japan; 2 Seikai National Fisheries Research Institute, Fisheries Research Agency, Nagasaki 851-2213, Japan; 3 Transdisciplinary Research Organization for Subtropics and Island Studies, University of the Ryukyus, Okinawa 903-0213, Japan

## Abstract

Hermaphroditism is ubiquitous among plants and widespread in the animal kingdom. It is an unsolved problem why reversed sex change has evolved in polygynous and protogynous reef fish. We have previously suggested that facultative monogamy occurs in low-density populations of polygynous species and that males that become single as a result of accidental mate loss may change sex when they meet larger males. In this study, to test this ‘low-density hypothesis', we conducted field experiments with the coral reef fish *Labroides dimidiatus* in which a portion of females were removed to create a low-density situation. The ‘widowed' males moved to search for a new mate when no male, female or juvenile fish migrated into their territories and paired with nearby single fish, whether male or female. Alternatively, males expanded their territories to take over the nearest pair whose male was much smaller. These results support our low-density hypothesis.

Hermaphroditism is ubiquitous among plants and widespread in the animal kingdom. For the evolution of sequential hermaphroditism, the direction of sex change in a species has successfully been explained, in relation to the mating system of the species, by the size advantage model^1^,^2^,^3^,^4^,^5^. For example, this model predicts that protogyny (female-to-male sex change) will be advantageous in polygynous fish in which large males monopolise mating with females. In such cases, only the largest females in a group can change sex (i.e., there is social control of sex change^6^). In the 1990s, however, the reversed sex change (male to female) was observed in certain polygynous and protogynous reef fish in the field or aquarium^7^,^8^,^9^. In natural populations, the reversed sex change has been observed in very few cases: there have been three examples in the goby *Trimma okinawae*^10^, two in the hawkfish *Cirrhitichthys falco*^11^ and one in the wrasse *Halichoeres trimaculatus*^12^. In contrast, reversed sex changes have been observed more frequently (n = 10) in the monogamous goby *Paragobiodon echinocephalus*^13^. Cases in which the reversed sex change was observed in natural populations seemed to occur after the loss of a mate, or of reproductive output, and a change in an individual's social status from dominant to subordinate after encountering a larger male^9^. However, field evidence is still very limited.

In polygynous groups, males seldom become widowed because the group contains multiple younger females. In low-density populations, however, facultative monogamy frequently occurs, even in polygynous species^14^,^15^. Low population density is known to affect various aspects of reproduction, including sexual patterns and mating systems. For example, monogamy will occur in low-density species because of a limited opportunity to encounter and sequester multiple mates^16^,^17^,^18^. If the opportunity to encounter another individual of the opposite sex is very limited, simultaneous hermaphroditism will be favoured^1^. This hypothesis is called the low-density model for the evolution of simultaneous hermaphroditism^1^. Similar situations may occur in low-density populations of a polygynous species for which populations with high or moderate density may be common elsewhere.

Therefore, we have previously proposed that the reversed sex change should be favoured in low-density populations of polygynous species^19^,^20^. In low-density populations in which facultative monogamy occurs, males may sometimes become single as a result of accidental mate loss. The widowed males will change sex when they meet a larger male in the vicinity because it is costly or risky to move farther when searching for a new mate of the opposite sex. To verify this ‘low-density hypothesis' for the evolution of reversed sex change, the following predictions (P) should be confirmed in the field: (P1) females will sometimes disappear from monogamous pairs, and males will become single; (P2) widowed males will wait or move to search for a new mate if no migration into their territories occurs, whereas they need not move if migration occurs; (P3) moving males will choose a new mate from among the nearest individuals, whether male or female, because of the cost or risk of farther movement; and (P4) when two males form a pair, the smaller will change sex to female according to the rule of social control (i.e., only the largest should be male^6^).

In previous field experiments, we removed all females from the study area in two polygynous coral reef fish, the cleaner wrasse *Labroides dimidiatus* and the rusty angelfish *Centropyge ferrugata*^20^. When no immigration occurred, a portion of widowed males moved to pair with other widowed males, and the smaller in the male-male pair changed sex. These results support two of the above predictions (P2 and P4). It was also suggested that the widowed males tended to choose the nearby male^20^, as is predicted in P3, but it has not been confirmed whether a widowed male would choose to pair with another widowed male even in the presence of females, since we removed all females in the previous study.

The situation created by removal of all of the females would seldom occur in the wild. Even in a low-density population, it is highly unlikely that all of the females would accidentally disappear at once. Widowed males may have a chance to choose a female that is part of a harem or a monogamous pair instead of another widowed male. Therefore, in the present study on the cleaner wrasse *L. dimidiatus*, we removed only a portion of the females; thus, approximately half of the males were widowed, and the remaining males were monogamous. The first prediction (P1) could be verified by comparing disappearance rates between the sexes in the monogamous pairs. The third prediction (P3) could be verified by examining whether the widowed males took over a female from the nearest monogamous pair, paired with the nearest widowed male, or moved farther to search for a single female.

## Results

**Figure 1:**
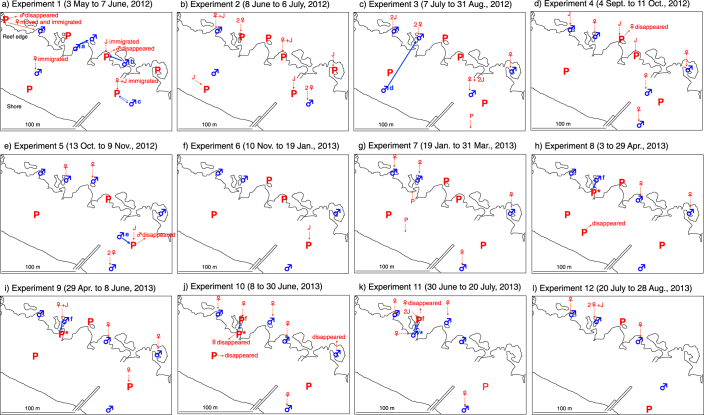
Map of the study area with the results of each experiment. Each map (a–l) shows the distribution of widowed males and monogamous pairs at the beginning of each experiment, their movement and disappearance and the migration into their territories over the course of the experiment. **♂**, widowed male; **♀**, female; P, monogamous pair; J, juvenile. Solid lines with an arrow indicate the movements of the widowed males or the female of a monogamous pair. Dotted lines with an arrow indicate immigration from unknown locations. The following males moved or expanded their territories. (a) ♂^a^ (78 mm TL) moved to the nearest widowed ♂ (84 mm) and began to change sex. ♂^b^ (76 mm) moved to the nearest widowed ♀ (65 mm), whose ♂ (70 mm) had disappeared. ♂^c^ (80 mm) tried to take over the nearest P (♂: 77 mm) but returned to his original territory. (c) ♂^d^ (82 mm) moved past the nearest P (♂: 78 mm) to the next nearest widowed ♂ (95 mm) and began to change sex. (e) ♂^e^ (80 mm) moved to the nearest widowed ♀ (71 mm) whose ♂ (ca. 90 mm) had disappeared. (h) ♂^f^ (100 mm) expanded his territory to the nearest P* (♂*: 70 mm). (i) ♂* (ca. 70 mm) of P* attacked his female, which had spawned with ♂^f^ (100 mm) three weeks prior to the attack. (j) ♂* (80 mm), whose female had disappeared, exhibited female spawning behaviours toward ♂^f^ (100) of P^f^. (k) ♂* (80 mm) completed a sex change to female and moved to the main cleaning station of ♂^f^ (100 mm) of P^f^, whose female had disappeared. The maps were drawn by T. Kuwamura using Microsoft PowerPoint from an aerial photograph (File no. COK-77-1 C24A 3) provided by the Web-mapping System (http://w3land.mlit.go.jp/WebGIS/index.html) of the National Land Information Division, National and Regional Policy Bureau of Japan.

The distribution of widowed males and monogamous pairs at the beginning of each experiment, their movement and disappearance, and the migration into their territories over the course of each experiment were determined for each of 12 experiments (Fig. 1). Only one of 56 widowed males (with the 12 experiments pooled) disappeared during the study period (Fig. 1j). Of the 48 experimental monogamous pairs (pooled over the 12 experiments), both mates disappeared from two pairs (Fig. 1h, j), the male disappeared from three pairs (Fig. 1a, e) and the female disappeared from three pairs (Fig. 1d, j, k). Thus, for monogamous pairs, the disappearance rate did not differ between the sexes (5/48 for both sexes; Fisher's exact test, P = 1.0).

Many immigrants were observed during the study period (Fig. 1), suggesting that the latent density of the study population was not low and that repeated removals were necessary to maintain the low-density situation. Female immigrants significantly preferred the territories of widowed males rather than those of monogamous pairs (Fisher's exact test, P = 0.002; Table 1), while such a preference was not found for juvenile immigrants (Fisher's exact test, P = 0.44; Table 1). The origins of the immigrating females and juveniles were largely unknown (most likely from outside the study area), except for one female who migrated from the nearest pair after her paired male disappeared (Fig. 1a).

When immigration occurred (n = 36; Table 2), the widowed males always accepted and paired with the immigrants, whether they were females (n = 37), juveniles (n = 8) or other widowed males (n = 2). The widowed males never moved when immigration occurred (n = 36), but a portion of the males moved (n = 4) or expanded their territories (n = 2) when no immigration occurred (n = 19; Table 2).

Movement of widowed males was observed in four cases (Fig. 1a, c, e). Two widowed males (76 mm and 80 mm) moved and paired with the nearest female (65 mm and 71 mm, respectively) whose mate (70 mm and approximately 90 mm, respectively) had disappeared; movements of these two males were approximately 25 m and 20 m distance, respectively (Fig. 1a, e). It is unknown whether the widowed males moved before or after the disappearance of the monogamous males; the monogamous male was 6 mm smaller than the widowed male in one case and approximately 10 mm larger in the other.

One widowed male (78 mm TL) moved approximately 25 m and paired with the nearest widowed male (84 mm) (Fig. 1a). They performed the upward swimming spawning behaviour with the smaller in the female position, i.e., the smaller male began to change sex.

Another widowed male (82 mm) did not take over the nearest pair, which was located at a distance of approximately 20 m (Fig. 1c), whose male (78 mm) was 4 mm smaller, but instead, moved to the next nearest widowed male (95 mm) located at a distance of approximately 80 m (Fig. 1c). The larger male exhibited courtship flutter-runs toward the smaller one, and the latter accepted the behaviour.

Expansion of territories was observed in two cases (Fig. 1a, h–k). One widowed male (80 mm) was observed to expand his territory temporarily to take over the nearest pair whose male was slightly smaller (77 mm) (Fig. 1a). The larger male repeatedly attacked the smaller one, which often fled from the attack; however, the larger male finally returned to his original territory, into which females later immigrated (Fig. 1b).

Another widowed male (100 mm) expanded his territory to the nearest pair whose male was much smaller (70 mm) (Fig. 1h). Approximately three weeks later, the larger male performed the upward spawning behaviour with the female (70 mm) of that pair. The larger male also exhibited courtship flutter-runs toward the smaller male, but the latter did not respond. The smaller male performed upward spawning with the female 5 min after the larger male left the pair's territory. In that case, the female spawned twice within 10 min but the presence of a gamete cloud was not confirmed owing to the presence of turbid water in both cases. Three weeks later, attacks by the smaller male against his mate were frequently observed (Fig. 1i). The smaller male sometimes left his territory and moved to the cleaning station of the larger male where cleaning occurred without aggression between the two males. Approximately three weeks later, the female of the pair disappeared, and the smaller male performed the upward spawning behaviour with the larger male, with the smaller male taking the female position (Fig. 1j). No gamete cloud was observed at that time, however, three weeks later the smaller male produced eggs that were confirmed by palpating its abdomen. The sex changing fish moved to the main cleaning station of the larger male whose female had disappeared (Fig. 1k).

## Discussion

The results of these field experiments on *L. dimidiatus* confirmed all four predictions (P1–P4), thus supporting the low-density hypothesis for reversed sex change in polygynous fish. P1 was proven by the observation of the disappearance of females from monogamous pairs. The disappearance rates in monogamous pairs did not differ between the sexes.

P2 was confirmed by the observation that the widowed males moved only when no immigration occurred. These males always accepted the immigrants, whether they were females, juveniles or other widowed males. Many immigrants were observed, and females preferred to immigrate into territories of widowed males rather than those of monogamous pairs, probably to avoid female-female competition^21^. In low-density populations, such immigration of females and juveniles cannot be expected, although immigration of other widowed males could be expected.

P3 was also verified because a portion of the widowed males moved to form pairs with nearby single fish, whether female (n = 2) or male (n = 2), or expanded their territories to take over a smaller neighbouring pair (n = 2). The widowed males were expected to choose nearby individuals for new mates to avoid the cost of farther movement; reducing movement costs has been suggested as an important factor for the evolution of bidirectional sex change^9^,^20^. The results of this study also suggest that male-male competition for females affects mate choice. Widowed males succeeded in taking over smaller pairs only when the size difference between the males was large enough (30 mm). When the size difference was small (3 mm), the widowed male failed to take over the pair. Moreover, one of the widowed males bypassed the nearest pair whose male was only 4 mm smaller and paired with the next nearest widowed male. These results suggest that widowed males avoided the cost of male-male competition when searching for a new mate.

P4 was confirmed in three cases where the smaller male began to change sex. The completion of sex change (as demonstrated by egg release) was confirmed in only one of the three cases. In the other cases, observations were discontinued due to the completion of an experimental cycle followed by the repetition of the removal experiment.

Our previous experiment in which all *L. dimidiatus* and *C. ferrugata* females were removed verified predictions P2 and P4^20^, whereas the present experiments in *L. dimidiatus* confirmed P1 and P3 also. In natural populations of polygynous fish, however, observations of reversed sex change have been very limited^10^,^11^,^12^. Although the sample size is small, the following examples seem to support the predictions (P1–P4) of the low-density hypothesis.

Three cases of reversed sex change in a natural population of the polygynous goby *T. okinawae* have been reported by Manabe et al.^10^. The authors observed four males that became single after the disappearances of their mates. Seven days after mate loss, one of these males, which had been monogamous, moved to a neighbouring polygynous group. The polygynous male was larger than the widowed male, which later changed sex. This example supports all four predictions of the low-density hypothesis. The remaining two cases of reversed sex change were non-functional because breeding as a male was not observed^10^. Two females became single, either through the disappearance of all other members of her group or by moving from a polygynous group to a vacant site; they underwent a sex change becoming male, as determined by the shape of the genital papilla, but could not acquire any mates and changed back to a female^10^. Therefore, only one case mentioned above was available to test the hypothesis.

In a relatively low-density population of the polygynous hawkfish *C. falco*, Kadota et al.^11^ observed two cases of reversed sex change in monogamous males whose mates had disappeared. The males changed sex and spawned with a neighbouring polygynous male that had expanded his territory. Whether the females disappeared before the males changed sex is unknown because the sex changes occurred during a six month non-observation period. In two other cases in which males became single after mate loss, the males acquired new mates and did not change sex^11^. These facts may also support the four predictions.

An example of reversed sex change in a natural population of the polygynous wrasse *H. trimaculatus*^12^ provides a somewhat different example from the above two species in which territorial males changed sex following mate loss. In the wrasse example, a small initial-phase (female-like colouration) male, which had demonstrated alternative reproductive tactics, changed sex when the population density decreased. It was suggested that a decrease in density at mating sites might have resulted in large territorial males successfully excluding small males from mating and subsequently caused the sex change of the small male^12^. This case may also suggest that the reversed sex change evolved in low-density populations of polygynous species as one tactic for acquiring new mates in widowed males.

Bidirectional sex change has also been observed in monogamous gobies that inhabit branching corals^9^,^13^,^22^,^23^. Mate-removal experiments, mimicking accidental mate loss, in *P. echinocephalus* revealed that widowed males and females moved to pair with nearby single fish of either the same sex or the opposite sex and that the smaller individual in male-male pairs and the larger in female-female pairs changed sex^24^. This situation in monogamous species is similar to that of facultative monogamy in low-density populations of polygynous species. Therefore, the most important factor for the evolution of reversed (bidirectional) sex change appears to be avoidance of the cost of movement (i.e., increased risk of predation) while searching for a new mate in low-density populations of both polygynous and monogamous species. The risk of movement has also been suggested as an important factor for the evolution of protandry (male-to-female sex change) in monogamous anemonefish that inhabit sea anemones with low population density^25^. In fact, in high-density populations of the anemonefish *Amphiprion clarkii*, it has been reported that widowed males rarely change sex but instead acquire a new female by her immigration or by their emigration^26^,^27^. Therefore, low density should be a key factor in the evolution of the male-to-female sex change in both monogamous and polygynous species. Although the low-density model was originally developed to explain the evolution of simultaneous hermaphroditism^1^,^28^, as opposed to sequential hermaphroditism, which may be explained by the size-advantage model^1^,^2^,^3^,^4^,^5^, our study suggests that the low-density model is also useful for explaining the evolution of the reversed sex change in polygynous and protogynous species.

## Methods

We conducted experiments in which a portion of female *L. dimidiatus* (Labridae) were removed from the coral reefs of Sesoko Island (26°39′ N; 127°57′ E), Okinawa, Southern Japan, in the same locality where the previous female removal experiments were conducted^20^. The study area was approximately 100 m × 220 m, off the Sesoko Station of the University of the Ryukyus (Fig. 1). Underwater observations and removals were conducted by snorkelling or SCUBA. Approximately half of the males were widowed by removing all females and juveniles from their territories, and the remaining males were made to be monogamous by removing all juveniles and females except the largest female. We repeated such removals at intervals of 3–10 weeks from May 2012 to August 2013, for a total of 12 sets of removals.

After conducting a preliminary census of the distribution of *L. dimidiatus* in the study area, we captured the males and all of the females and juveniles within their territories with hand nets and screen nets. The collected fish were anaesthetised with quinaldine diluted with seawater, and their total length (TL) was measured. The sex of each fish was determined by observing the shape of the urogenital papilla, by palpating the abdomen to release sperm or eggs^19^ and by the observation of courtship or spawning behaviour prior to capture. For individual identification, all of the males and those large females that would be used to form monogamous pairs were given a subcutaneous injection of coloured dye (Elastomer Fluorescent Tag, Northwest Marine Technology, Shaw Island, USA), and their body colour was recorded with a digital camera. After recovering from the anaesthetic, the males and large females were released at their collection sites; the remaining females and juveniles were released on other reefs separated from the study area. For the first experiment, six widowed males and six monogamous pairs were released (Fig. 1a); 3–6 widowed males and 2–6 monogamous pairs were released during subsequent experiments (Fig. 1b–l).

We observed the distribution of tagged and untagged individuals in the study area and the behaviour of the widowed males 3–10 weeks after each removal. Untagged immigrants were captured, and their TL was measured, sex determined, and body colour recorded (juveniles smaller than approximately 5 cm TL had different body colour from the adults). Immigrants were removed with the exception of any large females used to form monogamous pairs.

If a male-male pair formed after the movement of the widowed males, we observed courtship and spawning behaviour at the expected spawning time, usually after high tide^19^,^29^. Flutter-runs and body-sigmoids are courtship displays specific to male and female *L. dimidiatus*, respectively, and pelagic eggs are spawned at the top of an upward rush with the male straddling the female^29^,^30^. In one case, the individual that acted as a female in the spawning ascent was captured prior to the expected spawning time, and egg release was confirmed by palpating the abdomen. In the other cases, however, we started next experiment before confirmation of egg release to increase the number of observations for the prediction P3.

The research presented here complies with the current laws of Japan and the guideline of the Japan Ethological Society.

## Author Contributions

S.S., T. Kadota and T. Kuwamura contributed to the field experiments. T. Kuwamura took the lead in the data analysis and writing of the manuscript. All authors reviewed the manuscript.

## Figures and Tables

**Table 1:** Number of migrants into territories of widowed males and monogamous pairs. Data from 12 experiments were pooled

	Widowed males	Pairs
Number of territories	55*	40**
Number of immigrants		
Juveniles	8	9
Females	37	6
Widowed males	2	2***

*One male that disappeared was not included in this number.

**Eight pairs in which one or both mates disappeared were not included in this number.

***Expansion of territories by the larger widowed males; one expansion was only temporarily, with the male failing to take over the other territory.

Differences between widowed males and pairs: Fisher's exact test, P = 0.44 for juvenile immigrants, P = 0.002 for female immigrants.

**Table 2:** Relationship between the occurrence of migration into territories of widowed males and movement of those males. Data from 12 experiments were pooled

	Immigration	No immigration
Moved	0	6*
Not moved	36**	13

*Two cases of expanded territories and the other cases were complete movement of territories.

**For number of immigrants, see Table 1.
